# Fibroblast Derived Skin Wound Healing Modeling on Chip under the Influence of Micro-Capillary Shear Stress

**DOI:** 10.3390/mi13020305

**Published:** 2022-02-16

**Authors:** Sharda Gupta, Lavish Patel, Kunal Mitra, Arindam Bit

**Affiliations:** 1 Department of Biomedical Engineering, National Institute of Technology Raipur, Raipur 492010, India; sgupta.phd2016.bme@nitrr.ac.in (S.G.); patel.lavish14@gmail.com (L.P.); 2 Biomedical Engineering, Florida Tech, Melbourne, FL 32901, USA; kmitra@fit.edu

**Keywords:** shear stress, fibroblast, microchannels, wound healing, sandwich model, multilayered channels

## Abstract

Fibroblast cell migration plays a crucial role in the wound-healing process. Hence, its quantitative investigation is important to understand the mechanism of the wound-healing process. The dynamic nature of the wound-healing process can be easily implemented using a microfluidic-based wound-healing assay. This work presented the use of a microfluidics device to simulate traumatic wounds on fibroblast cell monolayers by utilizing trypsin flow and PDMS barrier. In this study, a microfluidic chip with a transparent silk film is reported. The placement of film provides 3D cell culture conditions that mimic a 3D extracellular matrix (ECM) like environment and allows real-time monitoring of cells. A numerical study was conducted to evaluate the influence of dynamic medium-induced shear stress on the base and wall of the microchannel. This could facilitate the optimization of the inlet flow conditions of the media in the channel. At the same time, it could help in identifying stress spots in the channel. The scaffolds were placed in those spots for evaluating the influence of shear forces on the migratory behavior of fibroblast cells. The in vitro microfluidic assembly was then evaluated for cell migration under the influence of external shear forces during the wound-healing phenomena. A faster wound healing was obtained at the end of 24 h of the creation of the wound in the presence of optimal shear stress. On increasing the shear stress beyond a threshold limit, it dissociates fibroblast cells from the surface of the substrate, thereby decelerating the wound-healing process. The above phenomena were transformed in both coplanar microfluidics surfaces (by realizing in the multichannel interlinked model) and transitional microfluidics channels (by realizing in the sandwich model).

## 1. Introduction

With increasing concern for skin disorders, the improved treatment of skin diseases such as a burn or skin tumor is essential. The use of animal models has the advantage of mimicking the complexity of the skin and its interaction with other organs, but it still has certain limitations like permeability of rat skin, hair follicles density, transappendagael absorption route, and most importantly, the ethical concerns related to the use of the animal model [1]. With an increase in the use of microfluidic technology, on-chip technology is frequently being used to create the in vivo condition [2,3]. The fabrication of artificial full-thickness skin equivalent, which represents native skin, has great importance for regenerative medicine. For the cultivation of a three-dimensional (3D) environment, microfluidic systems are essential to prepare skin grafts with uniform cell distribution. The evolution of the skin-on-chip model overcomes the limitation of static cultivation and hence increases the viability of the skin tissue model due to the dynamic cultivation of skin-related cells at in vitro conditions. In the static condition, nutrients and oxygen distributions depend only upon diffusion, and hence a small tissue-engineered construct can be successfully cultivated. However, in dynamic cultivation, large tissue-engineered grafts can also be cultivated.

Any disruption in the integrity of the skin due to mechanical trauma, thermal shock, and other diseases leads to the formation of wounds. Cell migration plays an important role in the wound-healing process. Fibroblasts cells migrate around the wound edge to allow contraction of the wound [4,5]. The influence of shear stress on the migratory proliferation of fibroblasts cells for wound-healing purposes was primarily examined on a macroscopic level [6,7]. Various wound-healing assays are available for the evaluation of cell migration. They include the creation of cell-free regions for the study of cell migration with mechanical wound assay, physical barrier assay, chemical barrier assay, electrical shock-induced wound, and optical ablation. Mechanical wounding, due to its simplicity and cost-effectiveness, is most commonly used. It can be classified into stamping and mechanical scratching assay. Physical barrier assay employs the use of a physical barrier or liquid barrier to block cell attachment to the substrate followed by removal of a barrier to initiate the cell migration process. The use of physical barriers leads to achievable wounds and minimal damage to cells. Various solid barriers are teflon fence, fleciperm disc, or microstensil. In wound-healing assay using a chemical method, wounds are created using chemicals such as sodium hydroxide or trypsin.

The various in vitro skin models commonly studied are wound-healing assay, 3D endothelial cell sprouting assay [8,9], diffusion study, toxicology study, efficacy test, anti-aging effects, shear stress study, drug development [10,11]. In vitro skin models are adopted for monitoring skin health using microsensors [10,11,12]. Post wound, the four classic stages of wound healing are hemostasis, inflammation, proliferation, and remodeling. The cytokines and growth factors released as inflammatory responses attract fibroblasts into the wound site. Fibroblasts from the dermis and nearby tissue migrate towards the wound bed. They differentiate into contractile myofibroblasts, which in turn, along with fibroblasts, produce an extracellular matrix responsible for cell growth and wound contraction. Inhibition of fibroblast migration may lead to impaired wound healing due to the absence of wound-healing signaling cascades. With the creation of a void, the migration of cells towards a cell-free area was monitored [13]. Tefft and co-authors had studied the dynamics of endothelial and fibroblast cells in the context of wound healing by creating a wound using a diamond knife [14]. Flow-induced shear stress has initially been used to study the effects of this stress on cell adhesion, mechanics, morphology, and growth in early microfluidic cell culture systems [15]. Recent studies are focused on reproducing physiologically relevant shear stresses to understand their effects in specific tissues and organs. The most important parameter which is part of the cellular environment is the mechanical forces that affect the differentiation, proliferation, phenotype, migration, and apoptosis of cells [6]. These forces provide signals for morphogenesis during wound healing. Skin is exposed to forces (compression, tension, and shear) at the cellular level due to tension forces from neighboring cells or surrounding ECM and contractile force due to the cytoskeleton of the cell [7]. The cells and tissues sense these forces by mechanosensing and convert them into a biological response by the process of mechanotransduction [8]. The microfluidic chip is nowadays used to induce mechanotransduction phenomena by inducing fluid flow and associated shear stress [16]. When the cells are exposed to fluid flow, shear stress is being induced at the periphery of the cells, resulting in increased cellular differentiation, cell metabolism, and cell barrier tightness [17].

As mechanical loading plays a significant role in skin wound healing, mimicking the concept of mechanical loading of skin has been widely used in skin tissue engineering by perfusion cultures. Wurfer and co-author had investigated the integration between keratinocytes, fibroblasts, and endothelial cells. This model was prepared to study inflammation, edema, and therapeutic drug testing [2]. Realizing the mosaic model of skin tissue is a typical interlinked bridge network formulation in the spatial domain. Hence, partial barrier-based substrates will help in integrating disintegrated cell types on a coplanar platform. Therefore, the skin wound model can be realized by two methods primarily: the first one considers multilayered channels with interconnected bridges. In first configurations, cells should be able to differentiate on the basis of their seeding destination while migrating from one cellular domain to a neighborhood cellular domain through interconnected bridges and thus should be able to establish continuity between subsequent layers of cells. The second method includes the development of a sandwich model. In this model, cells were introduced from different directions (perpendicular to a planar surface, tangential to lateral surfaces: left–right and frontal–rear) on a single sandwich substrate. Then, the migratory behavior of these cells was observed to evaluate the integral properties of the cellular clusters to be heterogeneous or homogeneous.

Due to presence of fluidic movement and fluid shear stress, classic microfluidic chip represents a noticeable improvement in physiological relevance when compared to traditional static cell culture. In spite of those advantages, many microfluidic devices exist as two-dimensional at the cellular level because the cells are still grown on a planar surface. In this paper, we presented a novel work by incorporating a reproducible silk-based film and physiologically relevant flow conditions. The two designs considered here consist of two components: a scaffold embedded within a PDMS microfluidic chip into which fibroblast cells were cultured to assess device efficacy.

The mechanism of the wound-healing process involves a complex dynamic environment consisting of the active participation of many mechanical and chemical factors. The mechanical stimuli of the flow over the wounded bed are some of the most contributing factors for effective healing. Hence, the evaluation of the influence of this effect was presented in the current study. A numerical model of the microfluidic platform was evaluated for optimizing the design of the microfluidic parameters and their different physical boundary conditions. The skin-on-chip model will be simulated with the dynamic microenvironment under continuously perfused different physiological fluid flow conditions. The culture of cells inside microfluidic channels enables the cell culture multiplexing using different skin-related cells. The artificial wound on the microfluidic cell culture scaffold was introduced by mechanical stumping and physical barrier. The film of scaffold could be placed at a stress rich location inside the microchannel to inject structural and biochemical niche while regulating cellular content for creating the desired microenvironment. A PMMA-based support layer was employed as a base chip to house the electrospun film. Wound closure was evaluated using optical microscopy, and cell migration were measured using ImageJ software. Both numerical and experimental studies were conducted hypothesizing that homogeneous flow velocity of media throughout the entire microfluidic chip can reciprocate the mechanotransduction behavior of migratory cells for faster wound healing in the presence of silk fibroin scaffold. The novelty lies with the incorporation of the wound over scaffold fixed within the microchannel. It makes the model closer to a realistic in vivo model. No other study has evaluated the multidimensional response of migrated cells at the wounded area over the scaffold and in the presence of controlled shear forces within microchannels.

## 2. Methodology

### 2.1. Designing of Device and Numerical Model

**Figure 1:**
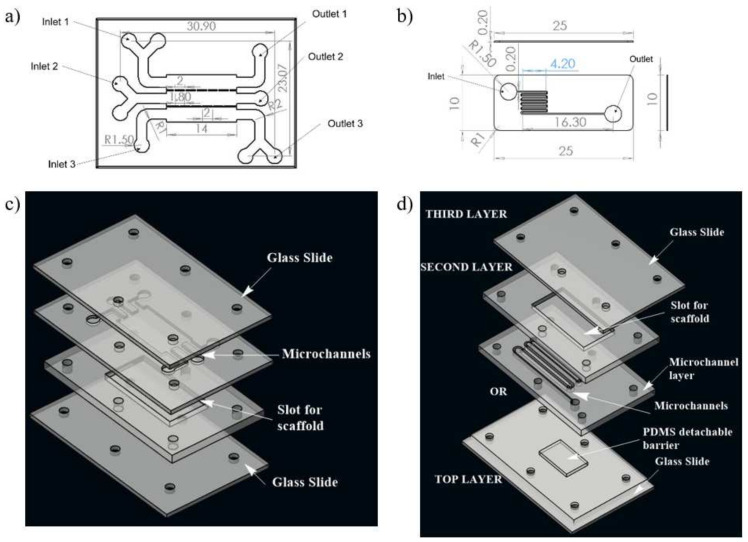
Schematic illustration of the two designs of microchannel of microfluidic chip used for the study; (**a**) design 1 with three inlet and outlet, (**b**) design 2 with one inlet and one outlet for fluid flow, (**c**) 3D view of design 1, (**d**) 3D view of complete chip of design 2. The media was injected through the inlet. In case of design 1, wound is made through flow of trypsin enzyme, whereas in design 2, the wound is created due to presence of PDMS barrier.

A numerical study was conducted for determining the shear stress distribution in microchannels of two different microfluidic networks, realizing two different approaches of synthetic wound creation. This network design was further fabricated to develop a microfluidic system for the development of an in vitro wound model. Computational fluid dynamics (CFD) was used to model flow and shear stress followed by a shear stress optimization using ANSYS Fluent 14.5 software. The geometries of two different microfluidic network designs for skin wound-healing modeling are illustrated in Figure 1.

Design 1 (coplanar multilayer model) consists of a housing for wound-healing assay. The chemical method (introducing trypsin) of wound creation was formulated for this model in the experimental phase of the study. In this model, three inlets were provided for cell seeding, chemical (trypsin) feeding for wound creation, and medium influx for the healing process, respectively (Figure 1a).

Design 2 (sandwich model) consists of housing for a detachable substrate on the bottom layer microfluidic circuit, which will act as a physical barrier for creating a wound. This design consists of three layers. There is a cover layer on the top of the first layer. The first layer is complementary to the second layer, facilitating recursive attachment and detachment of both layers. The second layer is a fluidic layer. Fluid flow evaluation on microfluidic circuitry for the second design was presented on this layer (Figure 1b). The geometrical configuration and wound-healing strategies of both designs adopted in the numerical study were tabulated in Table 1.

Computational fluid dynamics (CFD) simulations were conducted on co-planar models for both design 1 and 2 of microfluidic circuits. Quadrilateral mesh of fine size was performed using Pave meshing after conducting grid convergence study for coarse, normal, fine and finer mesh sizes. The fluid property of Dulbecco’ modified Eagle Medium (DMEM) was considered for the study. Fluid density was taken as 990 kg/m^3^ and viscosity as 0.0078 poise [18]. The inlet flow rate for both the designs were considered as 5 μL/min, 10 μL/min, and 20 μL/min.

The fluid flow was considered to be incompressible flow inside the channels. The flow profiles and shear stress distribution in the microchannel were studied using the steady-state Navier–Stokes equation (Bit et al. 2014). The governing equations for mass and momentum and evaluations of the flowing media are given by Equations (1) and (2), respectively.


(1) ∇·v = 0

(2) $$\frac{\partial v}{\partial t}+v\cdot \nabla v=-\frac{1}{\rho }\nabla p+\frac{\mu }{\rho }\nabla^{2}v$$

The cell culture media within a microfluidics capillary act as a shear thickening fluid, and thus it can be considered as non-Newtonian. Power Law model was adopted to model non-Newtonian viscosity of the fluid. The values of various parameters under normal conditions of DMEM are k = 14.67 × 10^−3^ Pa-s, and n = 0.7755. The outlet pressure was considered as an atmospheric state (zero gauges). Semi-Implicit Method for Pressure-Linked Equations (SIMPLE) formulation was used to solve the transient flow problem of viscous laminar flow.

### 2.2. Fabrication of Chip

Both the microfluidic designs were executed on a PMMA sheet. A CO_2_ laser cutter (GCC Laser Pro, Taipei, Taiwan) with an adjustable z stage was used to create the PMMA chip with microchannels. The design of the microfluidic chip was made on AutoCAD, and then, they were imported to CorelDRAW. The chips were fabricated using the movement of the laser along with x and y directions as per the CorelDRAW design. The chip architecture representing design 1 consists of three inlets, three outlets, and a scaffold that will be placed on the bottom layer, as illustrated in Figure 1c. In the upper layer of the chip, three channels were connected with interchannel bridges with respect to the middle layer, along with a U bend at each end to allow uniform distribution of media throughout the scaffold.

In the case of design 2, the first layer consists of PDMS mold to create a wound; the second layer consists of an inlet and outlet for fluid flow, and the scaffold will be placed into the chamber of the third layer. To optimize the uniform cell loading throughout the scaffold, channels were transformed into zigzag patterns. The presence of multiple zigzag patterned channels will give rise to hydraulic resistance within the microchannel.

**Figure 2:**
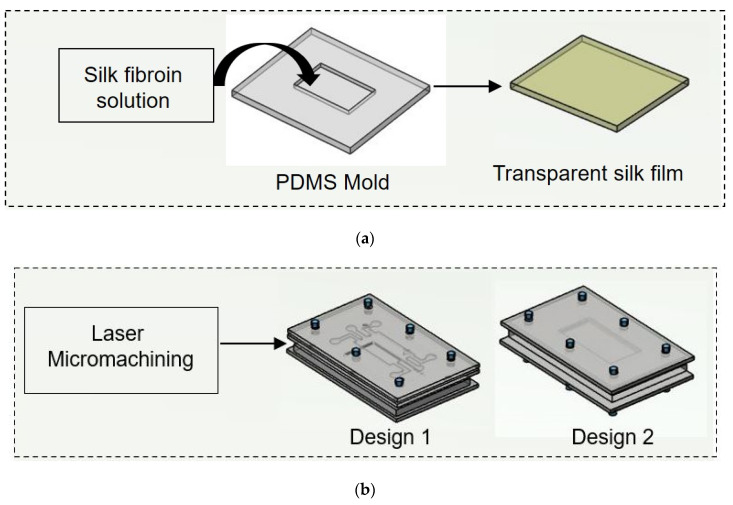
Schematic illustration of experimental procedure carried out. (**a**) Initially nonmulberry silk fibroin was added to PDMS mold to prepare transparent film. (**b**) Two chips were fabricated using laser micromachining.

In both designs 1 and 2, the lower layer consisted of a laser-ablated slot where a transparent silk film can be plugged in. The cell culture media flows over the scaffold within microchannels above the scaffold. To each inlet and outlet of the microchannel, Luer connectors were attached, and the interconnections were sealed using an adhesive (Loctite super glue) to prevent leakage. The layers were then clamped using mounting screws. The chip was used for wound-healing assay after plugging the scaffold into the slot provided for its placement. For ease of fabrication, a rectangular cross selection chip was considered. Fluids (cell culture media) were injected into microchannels by polyethylene tubing connected to a syringe pump for a controlled flow rate. Different flow rates (5 μL/min, 10 μL/min, and 20 μL/min) were maintained at the inlet of both the designed chips. The schematic of the experimental setup is shown in Figure 2.

### 2.3. Preparation of PDMS Mold

For the preparation of PDMS mold, Sylgard 184 silicone elastomer kit was used. The base and curing agent were mixed at a 10:1 ratio by weight. Both were mixed properly, followed by the removal of bubbles from the PDMS mixture. The resulting mixture was slowly poured on a 3D printed mold and kept inside an oven at 60 °C overnight. The cured PDMS was then removed from the 3D printed mold, cleaned with 70% ethanol to clean its surface and allowed to dry in a clean environment.

### 2.4. Isolation of Silk Fibroin

Silk fibroin protein was extruded from the glands of fully matured fifth instar larvae of nonmulberry Antheraea mylitta silkworms collected from the farm of Chhattisgarh Khadi Gram Udyog, Raipur, CG, India. Silkworm were carefully dissected and the gland was taken out and washed with deionized water. Silk fibroin protein was extracted by squeezing the posterior glands with fine tweezers. The water soluble sericin protein is removed and the obtained hydrophobic silk fibroin was dissolved further. The crude protein was dissolved in 1% sodium dodecyl sulfate (SDS) buffer containing TRIS (10 mM) and EDTA (5 mM) [19]. The protein solution was dialyzed against distilled water using a dialysis membrane (LA393, MWCO 12–14 kDa, Himedia) for 4 h.

### 2.5. Fabrication of Transparent Silk Film

Regenerated silk fibroin solution (3% *w*/*v*) was cast on the prepared PDMS negative mold (14 × 9 mm and 15 × 15 mm). The silk fibroin containing mold was then allowed to dry overnight by placing it inside the laminar hood with airflow. The obtained film prepared using the solvent evaporation method was treated with 70% ethanol to induce crystallinity. This allows the formation of a water-insoluble silk film due to the induction of β-sheet protein secondary structure [20]. Films were then washed with sterile phosphate buffer saline (PBS) before performing the in vitro study. Here silk film was used to enrich the ECM with amide binding sites for cell attachment.

### 2.6. Physio-Chemical Characterization of the Nanofibers

An attenuated total reflectance (ATR) accessory was equipped to Fourier Transform Infrared Spectrometer (FTIR, Bruker) to evaluate secondary structure of the prepared film. ATR measurement was performed in absorbance mode with 32 scans for each sample within the spectral range of 500 to 4000 cm^−1^. The FTIR of the samples helped to find out the functional group present in the composite film. The crystallinity analysis of the prepared film was performed using an X-ray diffractometer (PAN analytical, XPERT Pro). For XRD analysis, all samples were scanned [21,22].

### 2.7. Primary Cells and Cell Maintenance

This study was approved by the Institutional Ethical Committee (IEC) of the National Institute of Technology Raipur (Approval no-NITRR/IEC/2020/19). Mouse fibroblast cell lines (L929) were purchased from NCCS Pune. Briefly, the cell lines were maintained in high glucose Dulbecco’s modified Eagle’s media (DMEM; Gibco) supplemented with 10% fetal bovine serum (FBS; Gibco) and 1% penicillin-streptomycin (Gibco) in a humid atmosphere of 5% CO_2_, 37 °C and 95% RH. When the cells reach the desired confluence, they are harvested using 0.25% Trypsin-EDTA (Gibco) for further subculture or in vitro experiments.

Two microfluidic chip designs were used to determine the effect of flow rate and wound creation method. Instead of coating the cell culture channel in the chip with any extracellular protein, transparent silk film was placed inside the slot provided within the chip. The microfluidic chips were placed in the heating plate (37 °C) with a temperature controller. DMEM containing 10% FBS and 1% antibiotic was taken as test fluid. The test fluid was gently loaded into the microchannel and allowed for adhesion of cells in the absence of media flow. The test fluid was driven through the microfluidic channel at a flow rate of 5, 10 and 20 μL/min using a 10 mL syringe and syringe pump (HARVARD Apparatus, Ph.D. Ultra). A schematic of wound creation is shown in Figure 2. During wound creation, media were continuously perfused in the chips. Direct manipulation and physical exclusion method were employed to create wounds at in vitro study. In design 1, the growth medium is first injected through the device at a different flow rate (5 µL/min, 10 µL/min, and 20 µL/min) from the three inlets. The device is then injected with 0.05% trypsin solution in the middle inlet at a flow rate of 15 µL/min for 5 min. The well-controlled flow rates are achieved through syringe pumps. An inverted fluorescent microscope is used to track the wound creation within the channel in real-time. When fibroblasts detach from the substrate, the trypsin solutions are stopped, and the growth medium flow rate is increased for a minute to scrub away excess trypsin and to remove fibroblasts partially adhering to the substrate. In short, laminar flow patterns with growth patterns and trypsin are used to create the cell-free region (wound). In chip 1, initially, cell-containing media were flown from the two outermost layers and incubated at 37 °C, 5% CO_2_. Thereafter, microscopic images of the growing fibroblast cells were taken at the defined locations, which were visible through a bright field microscope. When the cell reached confluence in the two outermost layers, a wound was created along the middle channel. In design 2, the first layer consists of PDMS mold to create a wound. Through the second layer, fibroblast-containing media were flown. In the last layer, the silk scaffold was kept into which fibroblast cells were allowed to proliferate. When the cells reached confluence, the PDMS mold was removed to create a wound. Transparent silk film was considered so as to perform imaging of fibroblasts using Phase contrast microscopy (Axio Vert.A1 Bio, ZEISS). All studies were performed in triplicates.

For analysis of cell migration role in the healing of wounds, images were captured using an inverted microscope. The images were then processed using image analysis software ImageJ (National Institute of Health, Washington, DC, USA) to evaluate the gap between the wound edges. In this study, image analysis was used to focus the cell migration corresponding to different flow rates and the wound creation methods employed. For image analysis, the microfluidic chip was mounted on the platform of the microscope equipped with a camera.

### 2.8. MTT Study

Cell viability was measured by calorimetric estimation using 3-(4,5-dimethylthiazol-2-yl)-2,5-diphenyltetrazolium bromide (MTT; Thermofisher, Waltham, MA, USA) assay after seeding cells on silk film after 0 h, 12 h, and 24 h. After determined time points, cells seeded silk film were harvested and placed inside a 96-well plate, and 50 µL MTT solution (0.5 mg/mL in DMEM) was added to each well. The films were then incubated for 4 h at 37 °C. After incubation, the MTT containing medium was discarded and replaced by dimethyl sulfoxide (DMSO) to dissolve the blue formazan crystals. The absorbance was measured by microplate reader at 570 nm.

### 2.9. Statistical Analysis

All experiments were carried out in triplicates. The experimental data presented were expressed as mean, standard deviation. Statistical calculations and analysis were performed by comparing the data between groups and within groups by one-way analysis of variance (ANOVA) using Tukey’s test using Origin 2019 software.

## 3. Results

**Figure 3:**
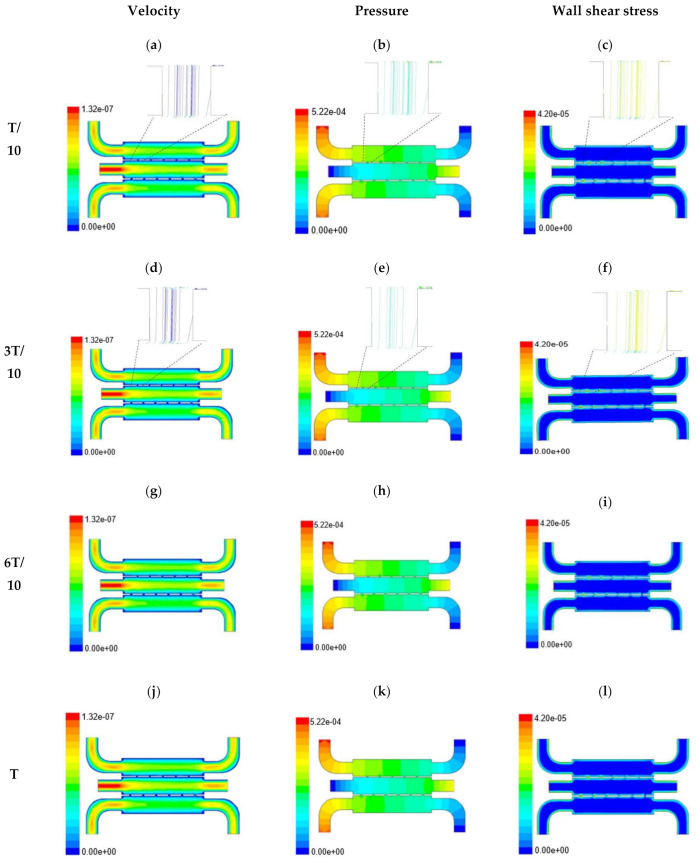
Numerical simulation of fluid flow inside the chip design 1 at flow rate of 5 µL/min. The different time step was taken to understand the profile of velocity (**a**,**d**,**g**,**j**), pressure (**b**,**e**,**h**,**k**) and wall shear stress (**c**,**f**,**i**,**l**) along the channel.

**Figure 4:**
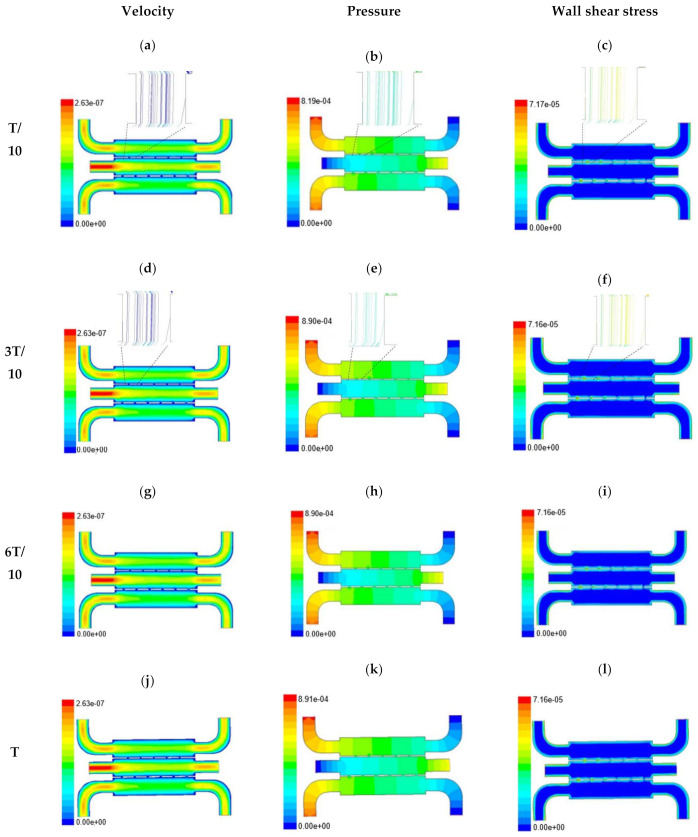
Numerical simulation of fluid flow inside the chip design 1 at flow rate of 10 µL/min. The different time step was taken to understand the profile of velocity (**a**,**d**,**g**,**j**), pressure (**b**,**e**,**h**,**k**) and wall shear stress (**c**,**f**,**i**,**l**) along the channel.

**Figure 5:**
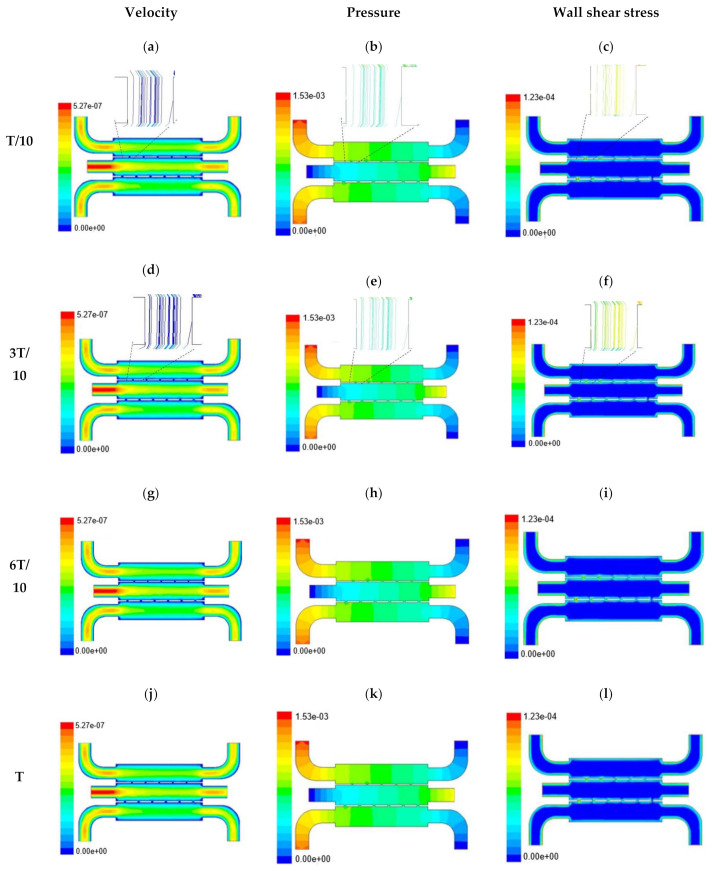
Numerical simulation of fluid flow inside the chip design 1 at flow rate of 20 µL/min. The different time step was taken to understand the profile of velocity (**a**,**d**,**g**,**j**), pressure (**b**,**e**,**h**,**k**) and wall shear stress (**c**,**f**,**i**,**l**) along the channel.

In this section, numerical evaluation followed by experimental evaluation was presented for the measurement of wound healing at in vitro condition under the influence of external shear forces. Figure 3, Figure 4 and Figure 5 show the velocity, pressure, and wall shear stress plots for fluid flow inside the microfluidic chip of design 1 at different inlet velocity of 5 µL/min, 10 µL/min, and 20 µL/min, respectively. From the Figure 3, it can be depicted that the magnitude of overall velocity stream function increases with time, particularly at inlet 2. It is primarily because of the trysin injection phenomena adopted to this channel. On the other hand, pressure profiles had shown increased pressure at the downstream of channel 1 and 3, whereas pressure builds up in upstream of channel 2. It leads to the development of differential pressure across channels, resulting in high velocity and pressure profiles in the bridges between adjacent channels. The magnitude of wall shear stress can be found to play a critical role in the migratory issue of media transportation across channels through bridges with the progress of time (as shown in Figure 3).

With the increase in inlet velocities, it was observed that the recirculation length (dead zones) near the wall (corner) at the upstream of all the channels decreases (as depicted from Figure 4 and Figure 5). At the same time, the pressure at the inlet was found to build up earlier in the second channel, while the remaining two channels had shown a decrease in dynamicity of the build-up pressure at the downstream of the respective channels (as shown in Figure 4). On the other hand, co-axial pressure on the lateral wall was found to increase with the development of the flow at a higher Reynolds number (as shown in Figure 5).

In the case of shear stress distribution, it was found that channels with mid-level flow profiles of the media had exhibited optimum shear stress near the interconnected bridges (as compared between Figure 4 and Figure 5). It had a significant influence on migratory cellular behavior for wound healing in experimental set up.

The time-dependent primitive variables data can be utilized to obtain the local wall shear stress, vorticity gradient, velocity variation profiles, and pressure profiles, along with the duration of flow. Analysis of the fluid-induced time-averaged flow was considered in the domain of non-Newtonian range in design 2. The local wall shear stress gradient had shown variation in the zig-zag folding (u-bend), whereas the value of wall shear stress reaches its peak. These values were calculated in the inner wall surfaces of all simulated cases. The highest value of local wall shear stress was found at the vertex of each bend as it assimilates to the higher-pressure ratio. The hydraulic area of flow (u-bend’s) for the vertex of each bend let the fluid travel a shorter distance, with a bulk rate of flow in the opposite direction, causing generation of higher local wall shear stress. It was necessary to create distribution and continuous flow of fluid uniformly to the receiver part (scaffold). A continuous flow of fluid sometimes creates irregularities in flow during propagation through different cross-sections such as channel or reservoir and builds a spinning motion along the wall of the channels that is commonly referred to as vorticity. Extremely low build-up of the spinning phenomenon was found maintained by reducing the Reynold’s number to absolutely low range. A minute amount of vorticity is compensated by the fluid-induced flow by diffusing onto the scaffold surface. Velocity and shear stress have an inverse relation that means as the shear stress increases, the velocity across the cross-section decreases. This situation certainly improves the interaction between the fluid and the scaffold. The immense flow of fluid creates a huge amount of shear stress across the wall of the channel, which in turn had slowed down the flow of fluid, creating a uniform gradient of velocity profile across the channel. This creates a significant gradient required to diffuse the fluid onto the surface of the scaffold. Centerline velocity was found to increase with the generation of progressive vortices with an increase in flow rate. Simultaneously, it was also found that pressure distribution was shifting from homogeneity to lower mean pressure distribution throughout the overall channel with an increased flow rate. It was also observed that wall shear stress magnitude had increased with an increase in flow rate primarily, but rate of increase of wall shear stress had ceased with further increase of flow rate beyond 10 µL/min. The range of the maximum and minimum pressure, velocity, and developed shear stress were tabulated in Table 2.

**Figure 6:**
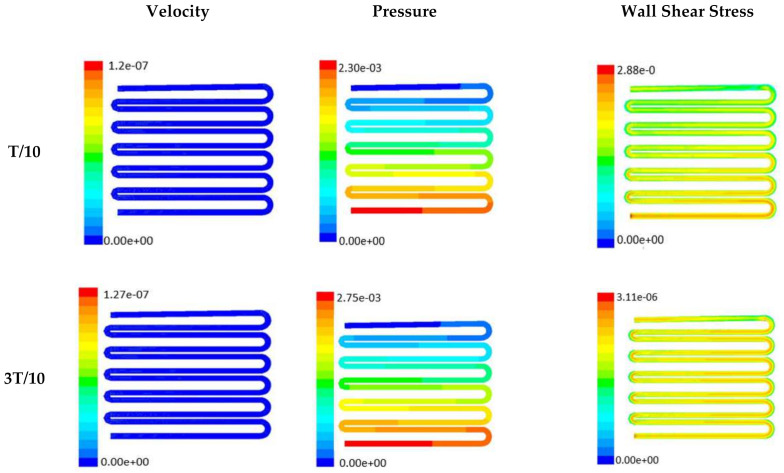

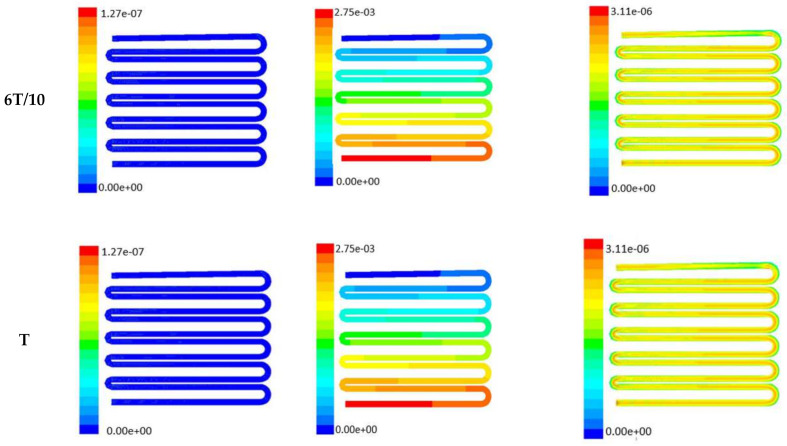
Numerical simulation of fluid flow inside the chip design 2 at flow rate of 5 µL/min. The different time step was taken to understand the profile of velocity, pressure and wall shear stress along the channel.

**Figure 7:**
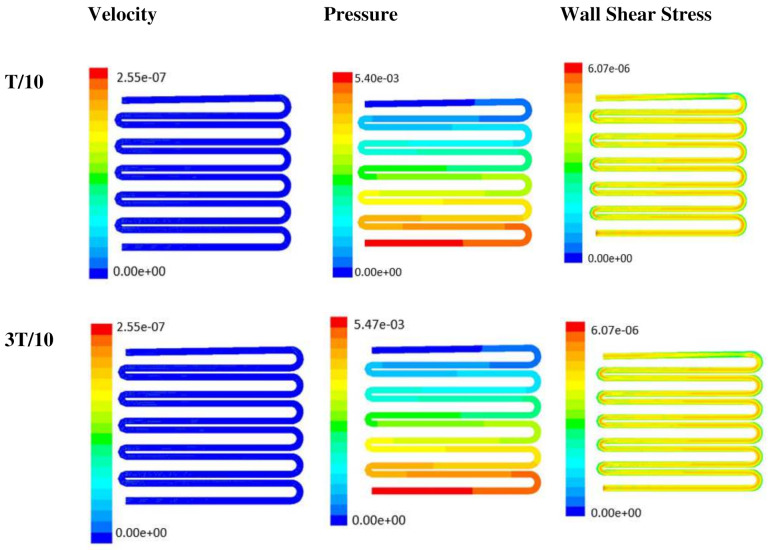

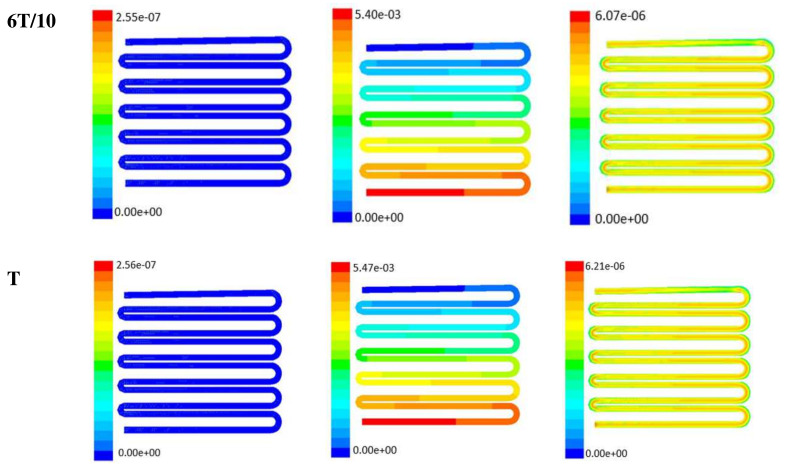
Numerical simulation of fluid flow inside the design 2 at flow rate of 10 µL/min. The different time step was taken to understand the profile of velocity, pressure and wall shear stress along the channel.

**Figure 8:**
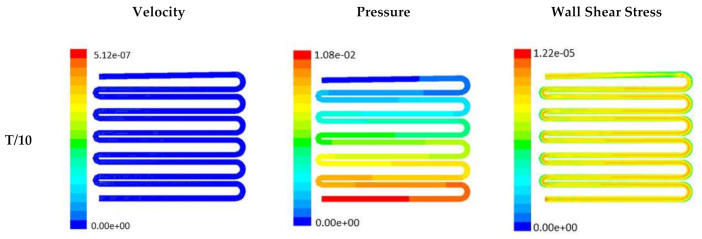

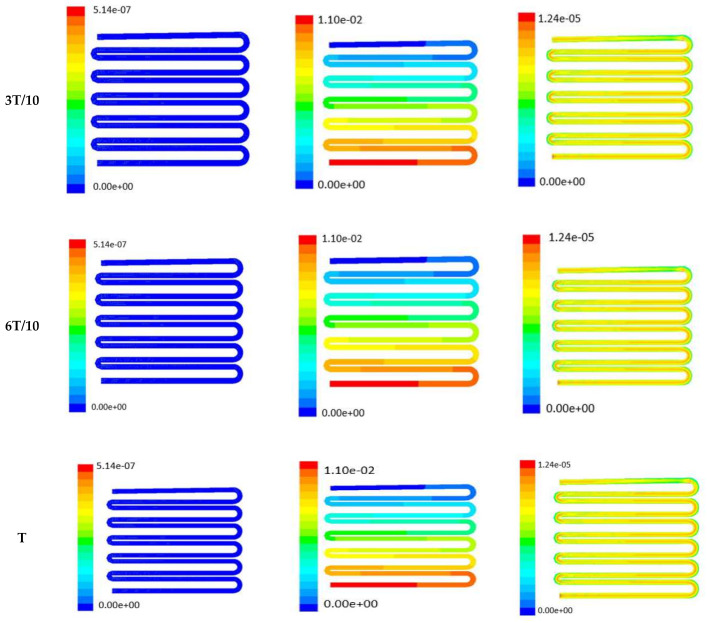
Numerical simulation of fluid flow inside the design 2 at flow rate of 20 µL/min. The different time step was taken to understand the profile of velocity, pressure and wall shear stress along the channel.

Time-dependent primitive variables evaluation for design 2 of the microfluidic chip is presented in Figure 6, Figure 7 and Figure 8 for the inlet flow rate of 5 µL/min, 10 µL/min, and 20 µL/min, respectively. Development of dynamic variation in velocity distribution was found highest in 20 µL/min flow rate (as shown in Figure 8), in comparison to 5 µL/min and 10 µL/min flow rate. It had witnessed the influence of momentum on the development of the flow in design 2 microfluidic chip. On the other hand, the maximum magnitude of pressure head was found constant for the last 70% of the flow cycle in case of inlet velocity of 20 µL/min flow. A linearity in the interdependent velocity and shear stress parameters can be observed for the inlet velocity of 5 µL/min to 10 µL/min, which was reaching a plateau for inlet velocity at 20 µL/min (as shown in Figure 6, Figure 7 and Figure 8). Variations in localized pressure for all inlet flow rates within the channels ensures homogeneous mixing of the media within the channel. It will further help in the process of adequate oxygen intake by the cells for their respective morphogenic activities. Further, a linear increase in shear stress-based stimulus (in form of mechanotransduction) to the cells will ensure proper signal transduction at intracellular regions. However, a plateau change in shear stress has minimal effect on the cellular signal transduction (as 20 µL/min flow rate shows plateau state of shear stress: from Figure 8). At the same time, it was observed that the magnitude of shear stress was maximum in channels with a flow rate of 10 µL/min at a magnitude of 1.24 × 10^−5^ s^−1^ (as seen in Figure 7). The same phenomena had been observed for design 1 chip (Figure 3). Thus, it can be concluded that the flow rate of 10 µL/min has been found as optimum to generate maximum effective wall shear stress due to abundant residual time of the fluid inside the microchannels of both designs 1 and 2 of the microfluidic chips.

Looking into the comparative study of the two designs of the chip, it was found that wall shear stress corresponding to 10 µL/min can be set optimal for inducing wound-healing phenomena. The following section has evaluated the validation of the numerical results.

**Figure 9:**
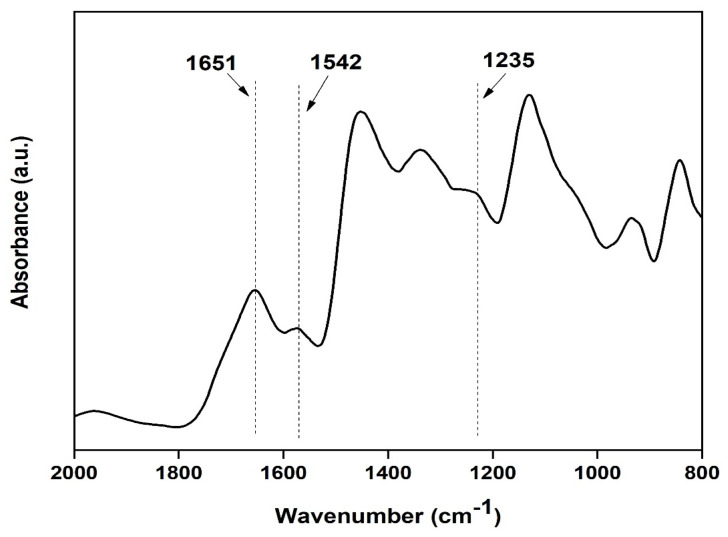
FTIR spectra of the silk fibroin casted transparent film.

Characterization of prepared silk film: FTIR spectra of transparent silk fibroin film in the range of 800 to 2000 cm^−1^ were obtained using Attenuated Total Reflectance (ATR) mode. The FTIR curves were characterized by absorption peaks at 1651 cm^−1^ (amide I), 1542 cm^−1^ (amide II), and 1235 cm^−1^ (amide III), confirming the presence of the various secondary structure of silk fibroin [20] (as shown in Figure 9).

**Figure 10:**
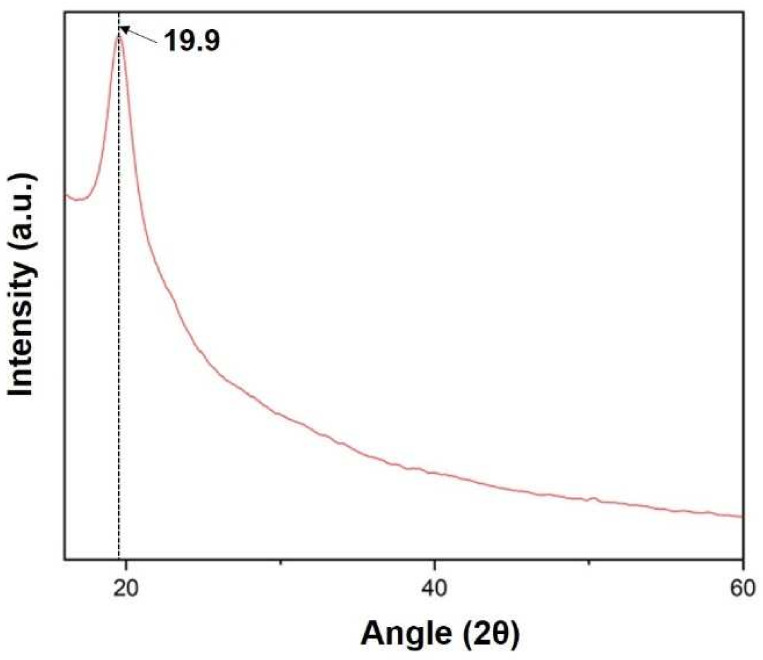
XRD spectra of the prepared silk fibroin film.

X-ray diffraction (XRD) of the sample was carried out at range 2θ = 10 to 60°. The XRD curve shows a peak at angle 19.9 confirming the presence of silk fibroin in the film, as shown in Figure 10 below [20].

When media was injected through two-side channels, an artificial wound was created in the middle layer. Wounds of limited (confined within the width of the microchannel) cross-sectional area were prepared due to dependency on media flow pattern for wound creation. Optimization of trypsin flow rate for the creation of the wound over the scaffold is tabulated in Table 3.

**Figure 11:**
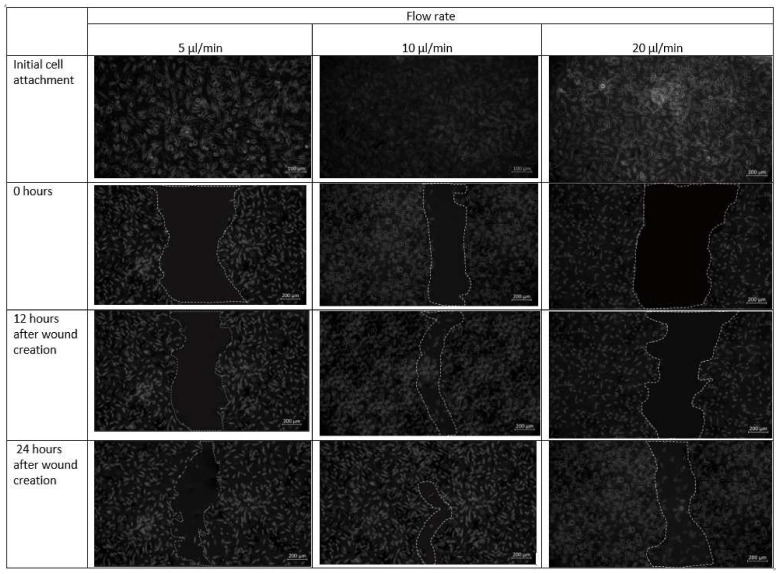
Micrographs of the wound-healing process of design 1 under a flow rate of 5 µL/min, 10 µL/min and 20 µL/min. Images are captured using bright field microscope (ZEISS Axio Vert.A1). Image analysis was performed using ImageJ software. Scale bar 200 µm.

Cell–cell and cell–matrix interactions are destroyed within minutes of trypsin administration, and the residual cell sheets are removed vertically from the substrate-creating wound (Figure 11). Over time, uneven spreading and migration of trypsin adds curvature to the leading edge of the ridge. The cell pattern in the design 1 is created in the midst of the confluent cell layer. Next, migration of cells towards the middle under different flow conditions is observed. The formed cell pattern that imitates cell arrangement in the event of a wound allows the study of cell migration during different physiological activities. Optical microscopy images of fibroblast cells cultured on a scaffold placed inside the chip design 1 after the introduction of the cell containing media are shown in Figure 11.

As observed in Figure 11, fibroblast cells were observed to be confluent in the scaffold placed inside the microfluidic chip. At a flow rate of 20 µL/min, however, a more discontinuous wound was formed due to substantial radial flow of fibroblast-containing media from the adjacent channels to the middle channel. After 12 h in culture, cells were able to migrate from the unwounded region to the wounded site and proliferated.

**Figure 12:**
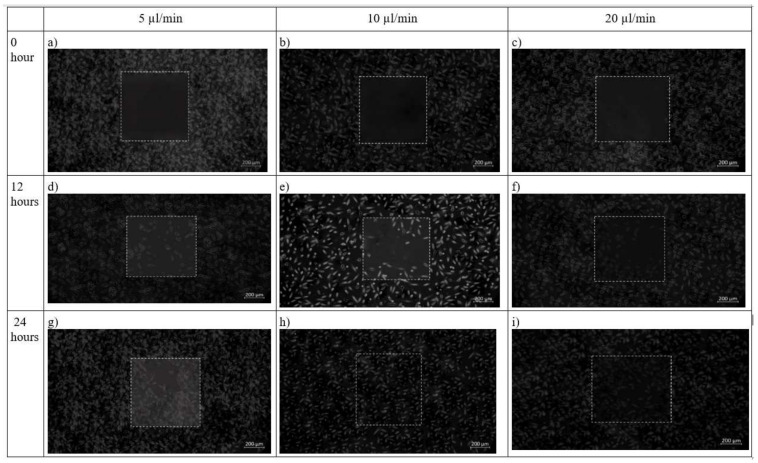
Brightfield phase images of the fibroblast cells under design 2 at 0 h, 12 h and 24 h in the silk film placed inside slot in the microfluidic device. Micrographs of the wound-healing process of chip 2 under a flow rate of 5 µL/min (**a**,**d**,**g**), 10 µL/min (**b**,**e**,**h**) and 20 µL/min (**c**,**f**,**i**) taken using ZEISS Axio Vert.A1 inverted microscope. Image analysis was performed using ImageJ software. Scale bar 200 µm.

In chip design 2, initially before cell seeding, the PDMS barrier was placed followed by removal of the barrier when a cell reaches confluence to provide an area for cell growth. After 12 h, cells had completely filled the wounded area while the flow rate was maintained at 10 µL/min. This was contributed by shear stress applied on the cells as shown in Figure 12. The net effect of wound healing due to cell migration is presented in Table 4. It can be depicted that in both the design of the microfluidic chip, the wound decreases with increase in time. Decremental rates of the wounded area were found higher in both chip design in the case of 10 µL/min flow profile of the media over the seeded cells on synthesized scaffold

**Figure 13:**
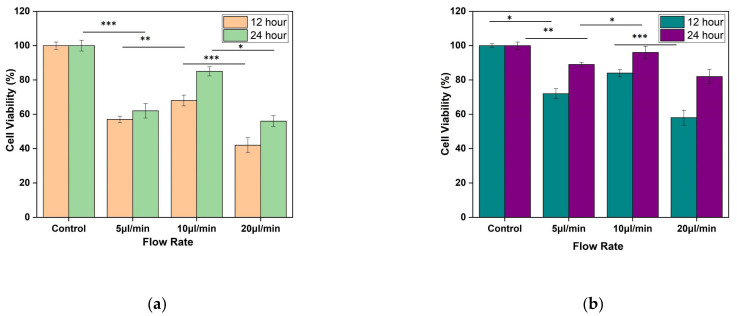
Result of MTT assay performed using L929 cell line. The fibroblast cell line was seeded on silk film and allowed to attach for 12 and 24 h of (**a**) Design 1, and (**b**) Design 2 migrating cells. The results are expressed as mean and error bars. The data was analyzed using One way ANOVA. Each experiment was performed in triplicate (* *p* < 0.05, ** *p* < 0.01, *** *p* < 0.005).

MTT assay shows the confluency of cells under shear stress at the wounded environment. It was observed that for both the designs, there is an improvement in cell confluency at the end of 24 h while the flow rate of the media was maintained at 10 µL/min (as shown in Figure 13a,b).

## 4. Discussion

The main aim of this study was to prepare a simple and effective wound-healing assay by using a microfluidic chip. In a conventional wound-healing experiment, cells were seeded and cultured on a Petri plate in a static condition, and then scratch was made on the confluent cells. These techniques lack the ability of reproducibility. Moreover, it has the disadvantage that, along with the removal of cells, it may harm the matrix on which scratch is made. In design 1, enzyme trypsin removes the cell layer but may leave residues that are not suitable for the growth of the new cells. Moreover, it demands proper trypsin from the chip after the creation of the wound. Design 2, the skin-on-chip approach, helps to prepare wounds in a convenient way without disturbing the matrix. Just with the removal of the PDMS barrier, a cell-free region will be created. These shape and cross-sectional area of the wound will be the same in each condition, allowing ease in observation and measurement of cell migration among different conditions.

Numerical and experimental data have shown that fibroblast cells could selectively migrate easily with the application of shear stress corresponding to a 10 µL/min flow rate. It has been observed that the change in shear stress with change in velocity was linear for inlet flow from 5 µL/min to 10 µL/min, whereas, for higher inlet flow of 20 µL/min, responsive shear stress achieved a plateau, with making insignificant contribution stimulus to the cells with shear stresses. To judge the accuracy of the numerical modeling approach in the present study, we compared our predictions using an experimental model. The frictional force of the media that acts tangentially on skin cells was considered as shear stress. It is influenced by the velocity, viscosity, and temperature of the media. Vorticity development with an increased flow rate in the type 2 design of the chip was due to the sinusoidal wavy pattern of the channels because it introduces Coriolis forces to the flowing media over the substrate. The presence of vorticity ensures the greater capability of the cellular integration in the spatial platform from multiple aspects of transport phenomena with the assistance of lateral flow and transitional flows.

Building a three-channel type 1 design can predict the modeling of the skin wound in a better way because the presence of one additional layer of channel form should assist in-depth creation of a wound on the superficial layer of the fibroblast. In type 1 design, the facilitation of substrate was limited to thin confinement for the successful creation of a wound scar. Hence, three-channel design of the chip would depict the integrity of the dermal segment in its healthy state, and the sandwich model can be more flexible for scar creation over the dermal layer at in vitro condition with a regulated micro-niche proliferative study of fibroblasts.

Fibroblast cells located at the periphery of the wound migrate towards the wounded area in the absence of any external factors such as growth factors, showing that the wound healing occurs in a natural manner inside the chip. With the application of stress, in general, healing speed increases. However, in conventional wound-healing assay performed in a Petri plate, the contribution of shear stress is missing. A change in flow rate within any microchannel will cause a change in shear stress on attached cells which can be easily monitored.

Two-channel designs were studied to determine the effect of shear stress associated with using the co-planar and transitional surface on fibroblast proliferation. Moreover, a standardized method of wound creation is employed in the sandwich model to enable reproducible wound size. This study includes the fabrication of a microfluidic device for quantitative investigation of cell migration on wound healing. The migration of fibroblast cells was monitored by capturing microscopic images over time. The generation of wound edges by use of the laminar flow of trypsin leads to the formation of wounds with uncontrollable shape and area.

The effect of different flow rates on the performance of fibroblast proliferation was studied. Figure 10 and Figure 11 display the impact of channel design and plane on fibroblast cells by using design 1 and design 2. At 5 µL/min, both designs 1 and 2 are effective. However, for a higher flow rate (20 µL/min), the flow of media containing cells diffuses towards the other channels and create wound of unknown dimension, whereas in design 2, with specific wound size, the fibroblast cells also did not migrate towards the wound. The shear stress induced in design 2 at 20 µL/min was 0.0387 Pa, which may not support the fibroblast cell migration. This can be justified by research that reported that shear stress induced by flowrate accelerates fibroblast migration, proliferation as well as differentiation in case of the damaged blood vessel. Moreover, shear stress between 0.05 to 25 dyne/cm^2^ helps in fibroblast cell migration [23]. Therefore, a flow rate of 10 µL/min was found to be more effective for fibroblast cell migration. The wound of predefined shape was consistently formed 0 when using design 2 at all flow rates. In design 1, wounds with run-to-run variations in shape and size were made.

The findings show that shear stress has a significant impact on the fibroblast cell migration mechanism. Cells migrate at comparable speed towards the wounded area under optimum shear stress.

## 5. Conclusions

Fibroblast proliferation and migration are vital in many biological processes, and they may be regulated through diverse microenvironmental factors. To explore fibroblast cell migration, a novel microfluidic-based cell migration assay is fabricated in this research. Two chip designs are exploited to generate cell patterns with almost consistent dimensions (design 1) and fixed dimensions (design 2). Using laminar flow patterning and PDMS barrier, the microfluidic system described in the research allows a cell pattern (wound) to be produced with consistent dimensions. In conclusion, it can be confirmed that a dynamic mechanotransduction phenomenon of cellular entities had always grown enhanced orchestra with the nonmulberry silk scaffold’s biochemical niche. Thus, it promotes faster wound healing by catalyzing the migratory behavior of cells towards the wounded region. In this study, two-chip designs were prepared: one with a central inlet employed with the flow of trypsin containing media and the second with a detachable post for wound creation to monitoring cell migration. The wounds created using a detachable PDMS barrier are reproducible and simple. Design 1 leads to the generation of wounds with high variability and poor reproducibility, whereas design 2 creates wounds with high precision and reproducibility. This dynamic wound-healing assay will provide more ease for carrying out research related to drug discovery, cellular pathway study, and wound-healing mechanisms. With an increase in the number of channels, more numbers of cells can be incorporated into the chip to study the dynamic and complex skin microenvironment.

## Figures and Tables

**Table 1:** Design Parameters.

Parameters	Description (Design 1)	Description (Design 2)
Wound-healing assay	Direct manipulation using trypsin	Physical exclusion using a PDMS mold
Device design	3 inlet, 1.50 mm radius of the inlet, 0.2 mm height, width, 3 outlet, 0.2 mm channel thickness	1 inlet, 0.2 mm height, 0.2 mm width, 1.5 mm radius of the inlet, 1 outlet
Impact of design on wound size creation	Nondeterministic shape of wound created	Deterministic and reproducible wound created

**Table 2:** Table showing maximum and minimum pressure, velocity and shear stress.

	Pressure (Pascal)		Velocity (ms^−1^)		WSS (Pascal)	
Amount	Maximum	Minimum	Maximum	Minimum	Maximum	Minimum
5 μL/min	283.661	0	0.01286	0.00833	0.0913	0
10 μL/min	830.21	0	0.0382	0.025	0.0182	0
20 μL/min	1362.722	0	0.06363	0.0417	0.0351	0

**Table 3:** Optimization of trypsin flow rate across silk film placed inside design 1 chip.

Trypsin Flow Rate	Time	Remarks
5 µL/min	4 min	No interface created between trypsin and media
10 µL/min	4 min	No interface created between trypsin and media
15 µL/min	4 min	Proper cleavage of cells with clear interface trypsin cells and media
20 µL/min	4 min	Cells were damaged and unstable trypsin flow was observed

**Table 4:** Table showing effect of cell migration at different flow rate inside design 1 and design 2. Zero hour is considered when a cell reaches confluency in case of design 1, whereas in case 2, it is the time when wound is created inside the chip.

Design 1 (Wound Area (in mm))				Design 2 (Healed Area (in mm))			
	5 µL/min	10 µL/min	20 µL/min		5 µL/min	10 µL/min	20 µL/min
0 h	619,042.843	536,347.072	881,491.515	0 h	516,127.637	512,156.844	515,587.264
12 h	529,054.573	283,111.059	705,521.213	12 h	421,749.287	262,697.214	242,967.842
24 h	407,404.345	161,375.886	523,519.110	24 h	74,976.281	22,441.196	103,555.969
